# Utility of transcranial magnetic stimulation in the assessment of spinal cord injury: Current status and future directions

**DOI:** 10.3389/fresc.2022.1005111

**Published:** 2022-10-05

**Authors:** Tarun Arora, Naaz Desai, Steven Kirshblum, Robert Chen

**Affiliations:** ^1^ Krembil Research Institute, University Health Network, Toronto, ON, Canada; ^2^ Department of Physical Medicine and Rehabilitation, Rutgers New Jersey Medical School, Newark, NJ, United States; ^3^ Kessler Institute for Rehabilitation, West Orange, NJ, United States; ^4^ Kessler Foundation, West Orange, NJ, United States; ^5^ Rutgers New Jersey Medical School, Newark, NJ, United States; ^6^ Edmond J. Safra Program in Parkinson’s Disease, Morton and Gloria Shulman Movement Disorders Clinic, Toronto Western Hospital, UHN, Toronto, ON, Canada; ^7^ Division of Neurology, University of Toronto, Toronto, ON, Canada

**Keywords:** spinal cord injury, transcranial magnetic stimulation, clinical assessment, neurophysiology assessment, international standards for neurological classification of spinal cord injury

## Abstract

Comprehensive assessment following traumatic spinal cord injury (SCI) is needed to improve prognostication, advance the understanding of the neurophysiology and better targeting of clinical interventions. The International Standards for Neurological Classification of Spinal Cord Injury is the most common clinical examination recommended for use after a SCI. In addition, there are over 30 clinical assessment tools spanning across different domains of the International Classification of Functioning, Disability, and Health that have been validated and recommended for use in SCI. Most of these tools are subjective in nature, have limited value in predicting neurologic recovery, and do not provide insights into neurophysiological mechanisms. Transcranial magnetic stimulation (TMS) is a non-invasive neurophysiology technique that can supplement the clinical assessment in the domain of body structure and function during acute and chronic stages of SCI. TMS offers a better insight into neurophysiology and help in better detection of residual corticomotor connectivity following SCI compared to clinical assessment alone. TMS-based motor evoked potential and silent period duration allow study of excitatory and inhibitory mechanisms following SCI. Changes in muscle representations in form of displacement of TMS-based motor map center of gravity or changes in the map area can capture neuroplastic changes resulting from SCI or following rehabilitation. Paired-pulse TMS measures help understand the compensatory reorganization of the cortical circuits following SCI. In combination with peripheral stimulation, TMS can be used to study central motor conduction time and modulation of spinal reflexes, which can be used for advanced diagnostic and treatment purposes. To strengthen the utility of TMS in SCI assessment, future studies will need to standardize the assessment protocols, address population-specific concerns, and establish the psychometric properties of TMS-based measurements in the SCI population.

## Introduction

### Spinal cord injury

Spinal cord injury (SCI) is a life-altering event with impairment of various neurological functions including motor, sensory, and autonomic dysfunction. These changes almost invariably result in a reduced quality of life. In the United States, SCI affects over 17,000 individuals each year and it has a prevalence of approximately 294,000 (1–3). The most common cause for SCI is motor vehicle crashes, and males account for 78% of new SCI cases (3). Recovery of upper and lower extremity function is a top priority for individuals with SCI (4, 5); however, the neurophysiological mechanisms underlying movement impairments are poorly understood (6, 7). A better understanding of the neurophysiological mechanisms underlying movement impairments and recovery can help in better prognostication and allow for more targeted and individualized therapies to improve motor recovery.

Following acute traumatic SCI, clinical examination remains the first and most important diagnostic approach to determine the extent of motor and sensory deficits, and the level and severity of injury, which can be used to characterize natural neurological recovery (8). The information gleaned from the examination and classification can inform the planning of rehabilitation strategies (9). In the chronic stages of traumatic SCI, usually defined as greater than 1-year post-injury, spontaneous recovery is rare; however, newer rehabilitation techniques (e.g., neuromodulation) are showing potential for neurologic recovery in individuals with chronic SCI (10, 11). There is a growing interest in advancing the use of electrophysiology (e.g., transcranial magnetic stimulation, somatosensory evoked potential, spinal reflexes) (12) and neuroimaging (diffusor tensor imaging, spinal tractography) (13) techniques to supplement the clinical assessments for characterizing residual connectivity and neurological recovery following SCI. Transcranial Magnetic Stimulation (TMS) is one of the non-invasive electrophysiology techniques that has been repeatedly proposed as a method to supplement clinical assessment in individuals with SCI (12, 14, 15). TMS-based measures assess the body structure and function domain of the International Classification of Functioning, Disability, and Health. Specifically, the TMS-based outcomes allow objective assessment of corticomotor neurophysiology to help monitor neurological changes following SCI.

In this review, we will briefly discuss the current best practice clinical assessment tools within the body structure/function and activity domains of the International Classification of Functioning, Disability, and Health and provide an in-depth review of the TMS-based measures that may potentially aid better prognostication and advance the understanding of neurophysiologic mechanisms underlying impairments and functional recovery.

### Clinical assessments after spinal cord injury

Clinical assessments in rehabilitation settings are often used to guide the progression of therapy post SCI. Several outcome assessment tools are designed to measure different domains under the International Classification of Functioning, Disability, and Health framework. Whereas these measures are important for devising patient's plan of care based on prognosis for recovery, the individual measures usually do not have any predictive value for long-term motor recovery. Clinical prediction rules (CPR) have been developed by researchers by combining clinical features, such as demographics, symptoms, physical examination findings, imaging results, and assessment scores (16–20). CPR may provide an estimate of the probability of the presence of disease (diagnostic CPR), the outcome (prognostic CPR), or response to treatment (prescriptive CPR) in a given patient (16, 17). A few prognostic CPR using logistic regression analysis have been developed in SCI, more notably to predict lower extremity/ambulatory recovery (18, 21–23). CPR may assist in planning for lifestyle changes, treatment decisions or help manage patient expectations and stratify patients for therapeutic intervention trials (24). There are some CPR methods that have prognostication value; however, they do not identify the neurophysiological basis of the prognosis of individuals with SCI (16). With a shift towards individualized treatment plans, it becomes important to identify individual patient's prognosis in the acute stages, so that treatment plans can be developed accordingly, and during chronic stages to monitor improvements with newer therapies targeted at neuro-restoration.

The Spinal Cord Injury Research Evidence (SCIRE) team published a standardized set of outcome measures developed in consultation with experts in SCI, for use in SCI clinical practice (25). This set consists of 32 measures that have been psychometrically validated in SCI population. Below we discuss some of the outcome measures that are commonly used both in clinical practice as well as in research in SCI population.

#### International standards for neurological classification of spinal cord injury

The International Standards for Neurological Classification of Spinal Cord Injury (ISNCSCI) in association with the American Spinal Injury Association (ASIA) Impairment Scale (AIS) is the most commonly used neurological examination and classification of severity of injury following traumatic SCI (8). The ISNCSCI has evolved over time with many revisions (26–28) and in its current form, offers clear instructions and consistent terminologies related to the level and completeness of SCI. The most recent revision from 2019 (28) incorporates two main changes including (a) a new taxonomy for systematic documentation of clinical judgment in the presence of non-SCI related conditions, and (b) a new definition of the zone of partial preservation, which applies not only to neurological complete but also to incomplete injuries with missing motor or sensory functions in the lowest sacral segments (28–30). The ISNCSCI is an impairment-based measure and assesses function in the body structure/function domain of the International Classification of Functioning, Disability, and Health. It involves sensory and motor impairment assessments in segments above and below the level of injury to define the neurological level of injury and the neurological “completeness” of injury based upon the sacral sparing definition. In addition, the motor and sensory scores are also used for stratification and prognostication purposes (14, 31). Although the ISNCSCI is the most widely used standardized clinical neurological assessment in SCI and has shown relatively good psychometric properties, there are challenges in its use for neurological classification and prognostication (30, 32), and its utility as a measure to estimate prognosis is debatable (33). The assessment is subjective and depends on the experience and training of the assessor (34–36). Similar to most clinical assessments, it relies on the participation of the patient and is affected by the heterogeneity of SCI (8). For example, given the subjectiveness of certain components of the scale, if sensory or motor scores are inaccurately assigned as zero for sacral sparing, then a patient classifies as AIS A (neurological complete injury) instead of AIS B (sensory incomplete), or AIS C (motor incomplete). Moreover, the scale has floor effects for AIS A and ceiling effects for mild injuries (i.e., AIS D) (32, 37). The ISNCSCI, most commonly the specific levels of injury (e.g., motor and neurological level of injury), upper extremity motor scores and AIS, are often used in clinical trials as an inclusion/exclusion criterion. Overall, however, AIS grades are rarely used as a sole outcome measure, likely due to the lack of sensitivity of this measure alone.

#### Upper and lower extremity clinical measures

Other than ISNCSCI, there are specific tools for the assessment of upper and lower extremity functional outcomes (Tables 1, 2). The SCIRE published a set of measures specifically designed for measuring upper and lower extremity assessment (25). Tables 1, 2 include psychometric properties of some of the upper and lower extremity measures that have been used in individuals with SCI. There are other outcome measures that were developed for other clinical populations, have excellent validity, and have been recommended for use in SCI population (e.g., berg balance scale, 10 MWT). It is beyond the scope of this paper to discuss all the clinical outcomes currently being used in SCI population. While these clinical measures have been validated for the assessment of upper and lower extremity function in individuals with SCI, they are still limited in terms of subjectivity, insufficient prognostic information, and lack of insight into the neurophysiological mechanisms.

**Table 1:** Clinical measures of upper extremity function and their psychometric properties in individuals with spinal cord injury.

Outcome measure	Primary construct	Psychometric properties
Spinal Cord Independence Measure version III (SCIM III)—self-care and grooming sub-scale	Independence level in ADLs, ICF domain: Activity	Inter-rater reliability: 71.6%–97.5% depending on item (100) Construct validity: Correlated with the Functional Independence Measure (*r* = 0.85) (100)
Capabilities of Upper Extremities Questionnaire (CUE)	Proximal and distal upper extremity function, ICF domain: Activity	Internal consistency: High (Cronbach's *α* = 0.96) (101) Validity: High Spearman's ρ correlation with the Graded Redefined Assessment of Strength, Sensibility, and Prehension measure *ρ* = 0.77–0.83 (102); high correlation with ASIA Upper Extremity Motor Score: *r* = 0.78 (103)
Graded Redefined Assessment of Strength, Sensibility, and Prehension (GRASSP)	Multidimensional—hand impairment and function, ICF domain: Body structure/Function, Activity	Inter-rater reliability: 0.84–0.96; test-retest reliability: 0.86–0.98 (102) Construct validity: 50% more sensitive than the ISNCSCI when defining sensory and motor integrity of the upper limb (102) Concurrent validity: significantly correlated with the SCIM, SCIM-self care, and CUE (102)
Grasp and Release test	Designed to measure function following FES and tendon transfer surgeries, assess lateral and palmar grasp. ICF domain: Activity.	Test-retest reliability is high for all 6 items (ICC = 0.87–1.00) (104, 105) Validity: statistically significant and moderate to high correlations between the 12-month Functional Independence Measure and the fork item (*r* = 0.624), the can item (*r* = 0.700) and the videotape item (*r* = 0.503) (104, 105)
Ashworth and Modified Ashworth	Used to assess spasticity in SCI, ICF domain- Body structure and function	Reliability: moderate inter-rater reliability (for MAS): ICC = 0.56 (106) Validity: moderate to high correlation of Ashworth (hip, knee, ankle) with SCATS (clonus, flexion, extension); moderate correlation between Ashworth (hip, knee, ankle) and Penn Spasm Frequency Scale (PSFS) (107)
Sollerman Hand Function Test	Designed to measure grips that are needed for certain ADLs and considers the quality and level of difficulty. ICF domain- Activity	Reliability: high inter-rater reliability (*r* = 0.98) (108, 109) Validity: high correlation of the Sollerman Hand Function test with the International Classification for Surgery of the Hand in Tetraplegia (Pearson's *r* = 0.88) and the Motor Capacities Scale (Spearman's *r* = 0.959) (108, 109)

ADLs, activities of daily living; ASIA, American spinal injury association; ICF, international classification of functioning, disability and health; MAS, Modified Ashworth Scale; ISNCSCI, The International Standards for Neurological Classification of Spinal Cord Injury; FES, functional electrical stimulation; SCATS, Spinal Cord Assessment Tool for Spastic Reflexes; SCIM, Spinal Cord Independence Measure version.

**Table 2:** Clinical measures of lower extremity function and their psychometric properties in individuals with spinal cord injury.

Outcome measure	Primary construct	Psychometric properties
Walking Index for Spinal cord injury II (WISCI II)	Amount of physical assistance, braces or devices needed to walk 10 m, ICF domain- Body structure\function	Reliability: very good inter-rater reliability (110); intra-rater reliability: >0.97 (111) Validity: concurrent validity: sensitive in patients with more impaired gait, however inferior to 6MWT and 10MWT in patients with good ambulatory function; poor correlation in patients with SCI who have poor ambulatory function (WISCI II scores <10); positively correlated with 10MWT, 6MWT, TUG (111)
Spinal cord injury functional ambulation inventory (SCI-FAI)	Gait is assessed in terms of gait parameters, assistive device use, walking mobility, ICF domain- Body structure\function	Reliability: inter-rater reliability: 0.703–0.840; intra-rater reliability: 0.850–0.960 (112) Validity: construct validity: gait score positively correlated with change in lower extremity strength (Pearson *r* = 0.58) (112); concurrent validity: highly correlated with the BBS (113)
Spinal cord assessment tool for spastic reflexes (SCATS)	Measures the primary spastic reaction in the SCI population, ICF domain- Body function	Reliability: high inter-rater and test-retest reliability (114) Validity: high correlation range with kinematic and electromyography = 0.69–0.94 (*P* < 0.01) (114)
Spinal Cord Independence Measure SCIM III: Mobility subscore	Measures the independence with transfers and household as well as community ambulation	Reliability: inter-rater reliability: adequate to excellent for the mobility in room and toilet transfers and excellent for the indoor and outdoor mobility (kappa values: 0.631–0.823) (115)

6MWT, 6-minute walk test; 10MWT, 10-minute walk test; BBS, berg balance scale; ICF, international classification of functioning, disability and health; TUG, timed up and go test.

### Transcranial magnetic stimulation-based assessment of spinal cord injury

**Figure 1:**
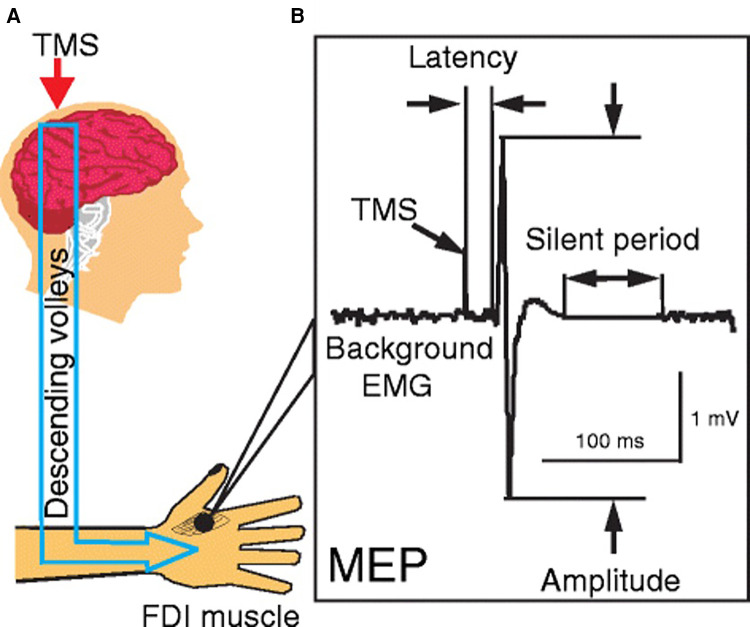
Single pulse transcranial magnetic stimulation. (**A**) Stimulation of primary motor cortex to activate corticospinal tracts and collection of responses from the first dorsal interossei (FDI) muscle; (**B**) characteristics of motor evoked potential and silent period—amplitude, latency, and silent period duration [Reprinted from Ni and Chen et al., 2015 (99); reprint permitted under Creative Commons Attribution 4.0 International License].

Transcranial magnetic stimulation is a non-invasive brain stimulation technique that uses a rapidly changing magnetic field to induce currents in the cortical structures (38). The induced current can depolarize the cortical neural structures and activate target muscles leading to motor evoked potentials (MEP) or inhibit the ongoing muscle activity to a silent period in the electromyographic (EMG) recordings (Figure 1). The MEP and silent period provide useful information on the excitatory and inhibitory pathways underlying motor impairments and recovery after SCI. The TMS-based metrics are associated with the extent of injury, and clinical-based assessment of impairments and activity (14, 39, 40).

#### Residual corticospinal connectivity

In the simplest form, TMS can detect the presence or absence of MEP in the muscles affected by SCI. Despite being diagnosed as a motor complete SCI, presence of MEP has been reported in the muscles with no detectable motor function, including abdominal, lower extremity and pelvic floor muscles (41–43). The presence of MEP in these muscles confirms residual connectivity in the corticospinal pathways, which may otherwise go undetected using clinical examination alone. MEP measurements can detect changes in the residual function and recovery of SCI over time. For example, a longitudinal study monitored MEPs from abductor digiti minimi muscles in 305 individuals with complete and incomplete SCI at 15 days, 1 month, 3 months, 6 months and 12 months following an acute traumatic SCI (C2-C8/T1; AIS A-E) (14). On the basis of MEP deterioration and evolution, the authors categorized MEPs as Abolished (absent in all assessments; 34%), Reappearing (absent initially, but consistent reappearance in at least 1/4 sessions; 25%), Inconsistent (occasionally present in at least 1/5 sessions; 4%), and Mildly (always present with normal latencies; 19%) or Severely deteriorated (always present with delayed latencies; 18%) (14). Out of these 305 individuals, ∼16% were diagnosed with clinically complete injuries (AIS A). Amongst those with AIS A, only 37% had fully abolished MEPs, remaining 63% had some presence of MEPs throughout the study. The findings suggest MEP can be present below the level of lesion, and in cases when absent, may evolve over time with spontaneous motor recovery even in those with clinically complete injuries.

The motor threshold, defined as TMS intensity (expressed as % maximum stimulator output; MSO) for eliciting consistent MEP, is typically higher in muscles impaired from SCI (40, 44–48). In some cases, the thresholds can be too high to elicit MEP even with the maximum intensity (i.e., 100% MSO). In some of these cases, there may be residual connectivity which may go undetected, leading to false-negative interpretation. In case of the absence of MEP with the 100% MSO, neurological reinforcement such as target and remote muscle contractions has been recommended to minimize the risk of false-negative interpretations (43, 49). For example, Williams et al. (2020) were able to obtain MEPs in pelvic floor muscles of all nine participants with chronic motor complete SCI (C6–T10 level) upon reinforcement using six different maneuvers involving isolated or combined contraction of abdominal, paraspinal, gluteal and pelvic floor muscle contraction. Another way of studying residual connectivity after SCI is through modulation of spinal reflexes by TMS (49–51). For example, TMS can facilitate plantar (50) or pudenda-anal reflexes (51), confirming preservation of descending pathways in some individuals with SCI. The above methods show that TMS is a useful tool to test the status of corticospinal tracts and other descending inputs in individuals with SCI. This is helpful in identifying “discomplete” SCI, which refers to clinically complete injuries with neurophysiological evidence of residual brain influence on spinal cord function below the lesion (52).

#### MEP latency and central motor conduction time

MEP latencies (time from TMS to the earliest deflection of the MEP) in individuals with SCI are typically delayed (Table 3). A longitudinal study evaluated MEPs in thenar muscles of individuals with SCI (C3–C7; AIS A–D) on multiple occasions from 19 to 1,109 days post-injury and found prolonged MEP latency throughout the follow-up period (45). Similar results have been reported in several studies for upper extremity (46, 53), lower extremity (39, 53, 54), and core muscles (42, 55) in individuals with SCI. Changes in the MEP latency are thought to result from axonal damage, demyelination and degeneration of the fast-conducting corticospinal tracts (56).

**Table 3:** Motor evoked potential latency in individuals with and without spinal cord injury.

Reference	Muscle	Test side	Number of participants	SCI (ms)	Healthy controls (ms)
Davey et al., 1998 (46)	Thenar Muscles	More Affected Side (lateralized symptoms) OR ELSE Dominant Side (Right)	SCI = 10 AB = 10	Rest = 27.7 (SE = 1.3) Active = 27.6 (SE = 1.3)	Rest = 21.3 (SE = 0.5) Active = 19.8 (SE = 0.5)
Alexeeva et al., 1998 (53)	Soleus	SCI: Stronger Side AB: Left Side	SCI = 10 AB = 20	42.5 (18.4)	34.0 (14.5)
Alexeeva et al., 1998 (53)	Abductor Hallucis	SCI: Stronger Side AB: Left Side	SCI = 10 AB = 20	48.2 (24.1)	38.2 (19.0)
Smith et al., 2000 (45)	Thenar Muscles	More Affected Side (lateralized symptoms) OR ELSE Dominant Side (Right)	SCI = 21 AB = 10	51–100 days post injury: Rest = 27 (SE = 1.2) Active = 26 (SE = 0.8)	51–100 days post injury: Rest = 21 (SE = 0.6) Active = 20 (SE = 0.5)
Barthelemy et al., 2015 (39)	Tibialis Anterior	More impaired side based on LEMS	SCI = 24 AB = 15	40.0 (6.0)	32.0 (2.0)
Squair et al., 2016 (41)	External/Internal Oblique	Right and Left side values collapsed	SCI = 13 AB = 13	23.6 (3.2)	21.8 (2.9)
Squair et al., 2016 (41)	Sartorius	Right and Left side values collapsed	SCI = 14 AB = 14	27.8 (7.1)	23.4 (2.5)
Squair et al., 2016 (41)	Rectus Femoris	Right and Left side values collapsed	SCI = 5 AB = 5	31.3 (6.9)	22.8 (1.7)
Squair et al., 2016 (41)	Tibialis Anterior	Right and Left side values collapsed	SCI = 1 AB = 1	48.3	33.3
Squair et al., 2016 (41)	Soleus	Right and Left side values collapsed	SCI = 2 AB = 2	52.7 (9.1)	34.5 (3.3)

AB, Able-bodied; LEMS, lower extremity motor score; ms, milliseconds; SCI, spinal cord injury; SE, standard error of the mean.

Although MEP latency is indicative of central and peripheral conduction, it can be combined with peripheral nerve conduction measurements to calculate the central motor conduction time (CMCT), which is an estimate of the conduction time of corticospinal fibres from the motor cortex and spinal motor neurons (57, 58). The CMCT is estimated by subtracting the spinal motor neuron to muscle latency (peripheral conduction time) from the cortex to muscle latency (MEP latency). The peripheral conduction time can be calculated by using M-wave and F-wave latencies that are elicited by stimulation of the peripheral nerves (57, 58). M-wave is an early response to peripheral stimulation resulting from a direct activation of the target muscle, whereas F-wave is a smaller and more variable later response resulting from activation of the α-motoneuron by the antidromic volley (58). 1 ms is the estimated turnaround time for the stimulus through the cell body of the spinal motor neuron (58) (see below Equation 1).


(1) $$\begin{array}{rl} & \mathrm{Central}\;\mathrm{Motor}\;\mathrm{Conduction}\;\mathrm{Time}\;\left(\mathrm{CMCT}\right)\\ & \;=\;\left(\mathrm{MEP}\;\mathrm{Latency}-\mathrm{Peripheral}\;\mathrm{Conduction}\;\mathrm{Tim}e^{*}\right) \end{array}$$

$$\begin{array}{rl} & *\mathrm{Peripheral}\;\mathrm{Conduction}\;\mathrm{Time}\\ & \;=\left[\left(M-\max\;\mathrm{latency}+F-\mathrm{wave}\;\mathrm{latency}-1\right)\right]/2. \end{array}$$

**Figure 2:**
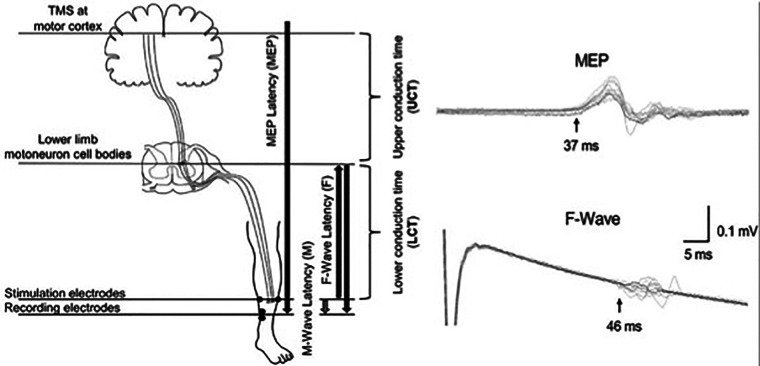
Calculation of the conduction times for paired associative stimulation. Use of upper conduction time (central motor conduction time) and lower conduction time (peripheral conduction time) for targeting the stimulation to lower limb motoneuron cell bodies. Use of latencies of MEP, F-wave and M-wave for precise calculations of the conduction times [Reprinted from Fok et al., 2020 (64); reprint permitted under Creative Commons Attribution License; CC BY].

CMCT is delayed in individuals with SCI compared to healthy controls (Table 4). A study found delayed CMCT for the first dorsal interosseous muscle in about half of the individuals (55/113) who had consistent MEPs (14). The CMCT values were delayed after acute SCI and remained delayed for at least 12 months (14). These findings confirm that TMS can be used to objectively measure the delay in conduction time of corticospinal fibres. TMS in combination with peripheral nerve stimulation has also been used to study the influence of afferent input on motor cortex excitability in individuals with SCI (54, 59). A study on 8 individuals with tetraplegia (C3–C7; AIS B–D) reported reduced short-latency afferent inhibition in the flexor carpi radialis muscle, which is typically seen in healthy subjects at ∼15–18 ms following median nerve stimulation (59). Another study in 22 individuals with SCI (C3–L5; AIS C–D) reported loss of MEP facilitation in the tibialis anterior muscle by prior (∼50–60 ms) conditioning stimulation of the tibial nerve, but intact facilitation with conditioning stimulation of the common fibular nerve (54). In addition, precise calculations of the CMCT have been used to design targeted paired-associative stimulation neuromodulation approaches to facilitate functional recovery after SCI (Figure 2) (60–64). The paired-associative stimulation approaches are based on the Hebbian principle of associative plasticity, i.e., “neurons that fire together, wire together” (65, 66). These studies support the use of TMS in developing highly precise and targeted non-invasive neuromodulation for rehabilitation.

**Table 4:** Central motor conduction time in individuals with and without spinal cord injury.

Reference	Muscle	Test side	Number of participants	SCI (ms)	Healthy control (ms)
Petersen et al., 2017 (14)	Abductor digiti minimi	Both Sides	SCI = 305	15 days = 7.47 (SE = 0.46) 1 month = 7.04 (SE = 0.35) 3 months = 7.09 (SE = 0.33) 6 months = 7.14 (SE = 0.36) 12 months = 7.23 (SE = 0.39)	Not applicable
Nardone et al., 2008 (74)	First dorsal interosseous	Both Sides	SCI = 1 AB = 10	Right = 6.1 Left = 6.4	Right = 6.1 (95% CI = 3.3) Right = 6.2 (95% CI = 3.6)
Bunday et al., 2018 (61)	First dorsal interosseous	SCI: Less affected AB: Right	SCI = 17 AB = 14	6.2 (1.5)	3.5 (0.8)

AB, Able-bodied; CI, confidence interval; ms, milliseconds; SCI, spinal cord injury; SE, standard error of the mean.

#### Corticomotor output and gain: MEP amplitude, area, and recruitment curve

The MEP amplitude (14) and area (48) are commonly used measures of corticomotor output. Typically, MEP amplitude is measured “peak-to-peak”, from negative to positive peak in EMG activity. However, some studies have measured amplitude from the baseline to the negative peak (14). The MEP amplitudes at a given TMS intensity (absolute and relative to motor threshold) are smaller in individuals with SCI compared to healthy controls (14, 59).

MEP amplitudes at multiple TMS intensities from subthreshold to suprathreshold levels result in sigmoid-shaped stimulus-response, input-output, or recruitment curve (48, 54, 67, 68). This curve may be plotted with Boltzmann function, and characteristics such as slope (rate of increase in MEP amplitude with increasing TMS) and highest MEP amplitude (MEP_max_) are evaluated. The amplitudes at the suprathreshold intensities including the MEP_max_ are smaller in individuals with SCI compared to healthy individuals in the affected upper (48, 68) and lower limb (54) muscles These studies show that TMS can be used to capture reduced corticomotor output in individuals with SCI.

#### Silent period

TMS can suppress ongoing muscle activities in the target muscles, causing electrical silence in the surface EMG (Table 5). This brief interruption can be observed at subthreshold (45, 46) and suprathreshold (40, 44, 69) intensities and is termed the contralateral silent period (cSP). The early part of cSP is thought to involve spinal inhibitory networks (70), whereas the later part involves intracortical circuits (71). The onset of cSP obtained using subthreshold TMS is delayed in muscles impaired from SCI compared to those without SCI (45, 46). A study found delayed cSP onset latencies in the thenar muscles of individuals with SCI (C3–C7; AIS A–D) over multiple occasions from 19 to 1,109 days post-injury (45). The authors suggested the delay in cSP onset latency to be reflective of reduced intracortical inhibition to facilitate movement recovery (45). However, the delay in cSP onset may also be reflective of changes in the early part of cSP, which involves the spinal inhibitory mechanisms (70, 72, 73). The duration of cSP is another parameter that changes following SCI. A study used suprathreshold TMS intensities and reported prolonged cSP in extensor digitorum communis muscle of 9 individuals with chronic SCI at C5-C8 level (40). There are different explanations for prolonged cSP duration after SCI. Firstly, SCI leads to impairments in the corticospinal tracts leading to higher motor thresholds, but the cortical inhibitory interneurons are spared. Use of suprathreshold TMS requires higher absolute TMS intensities (%MSO) that may activate more intracortical inhibitory neurons (40). Secondly, the loss of inhibitory afferent inputs due to SCI may lead to increased γ-aminobutyric acid (GABA)-mediated intracortical inhibitory activity (74). A study reported a loss of cSP in 3 individuals with clinically complete SCI (2 non-traumatic causes) (75). The authors argued that abnormal ascending impulses produced by the cervical cord lesions might have induced motor cortical hyperexcitability, resulting in loss of cSP (75). The changes in cSP have also been reported in muscles above the level of lesion (69). Another study reported prolonged cSP in the abductor pollicis brevis and biceps brachii muscles in six individuals with thoracic or lumbar level (one incomplete L1 lesion) injury (69). Since these muscles were above the level of lesion, the changes in cSP duration were due to reorganization of neural structures at a supraspinal level. The above findings show that the TMS-based silent period measurements provide an objective assessment of the inhibitory networks after SCI.

**Table 5:** Contralateral silent period latency and duration findings in individuals with and without spinal cord injury.

Reference	Muscle	Test side	Number of participants	Metric	SCI (ms)	Healthy control (ms)	CSP definition + findings
Davey et al., 1998 (46)	Thenar	More Affected Side (lateralized symptoms) OR ELSE Dominant Side (Right)	SCI = 10 AB = 10	Onset Latency	51.8 (SE = 1.8)	33.4 (SE = 1.9)	From the stimulus to the point in the record where the EMG fell consistently below mean background levels using sub-threshold TMS intensity
Smith et al., 2000 (45)	Thenar	More Affected Side (lateralized symptoms) OR ELSE Dominant Side (Right)	SCI = 21 AB = 10	Onset Latency	50 (SE = 2.2)	32 (SE = 1.5)	From the stimulus to the point in the record where the EMG fell consistently below mean background levels using sub-threshold TMS intensity + Reported values at 51–100 days post injury
Freund et al., 2011 (40)	Extensor Digitorum Communis	Dominant side (9 = right; 1 = left)	SCI = 9 AB = 14	Duration	Median = 130 (IQR = 60)	Median = 96 (IQR = 30)	From the time-point of TMS stimulus artefact to the resumption of sustained EMG activity + CSP duration negatively correlated with cervical cross-sectional area
Sfreddo et al., 2021 (44)	Abductor Pollicis Brevis	Side with lower motor thresholds and more consistent central and peripheral electrophysiological responses	SCI = 9 AB = 12	Duration	Median = 102.5 (IQR = 76.3–148.6)	Median = 95.4 (IQR = 86.6–110.2)	From the end of MEP to the earliest resumption of pre-TMS EMG activity. Differences between groups not significant
Nardone et al., 2008 (74)	First Dorsal Interossei	Both sides	SCI = 1 AB = 10	Duration	Right = 245 Left = 252	Right = 162.6 (95% CI = 82.2) Left = 165.2 (95% CI = 83.4)	From the end of the EMG response to the return of sustained poststimulus EMG activity

AB, Able-bodied; CI, confidence intervals; CSP, contralateral silent period; EMG, electromyography; IQR, Interquartile range; MEP, motor evoked potential; ms, milliseconds; SCI, spinal cord injury; SE, standard error of the mean; TMS, transcranial magnetic stimulation.

#### Cortical muscle representations

There is spontaneous and treatment-induced corticospinal reorganization following SCI (see reviews by Brown and Martinez, 2019 (76); Oudega and Perez, 2012 (56)). TMS-based motor maps have been used to study the cortical reorganization following SCI (40, 77–81). For example, the center of gravity (COG; the region thought to approximate the location of the highest density of corticospinal projections) of the cortical map for extensor digitorum communis muscle shifted posteriorly towards the hand representation in the anatomically defined hand knob in the central sulcus in individuals with chronic SCI (40). A case study in an individual with a transient (lasting ∼5 h) episode of complete SCI at the C5 level found a posterior shift of the COG for another hand muscle (abductor pollicis brevis) at 1-day post-injury (79). Interestingly, there was a partial reversal in the shift of COG within 10-days of the injury, and complete reversal at a 2-year follow-up that corresponded with functional recovery (79). The changes in motor map from the resting state to an active state (during voluntary contraction) also differ in individuals with SCI in comparison to healthy controls. A study of 22 individuals with chronic SCI (C2–C8; AIS A–D) found the motor map area reduced upon voluntary contraction of the target muscle (first dorsal interossei) and other proximal muscles (biceps brachii), whereas in healthy controls map areas increased upon contraction of the same muscles (81). Another study reported smaller motor map areas for severely impaired (motor power 1/5) forearm muscles in three (out of 10) participants with chronic SCI (C4–C6; AIS A–C), whereas the remaining seven participants had values comparable to healthy controls (47). The authors suggested that severely impaired muscles with normal motor maps (along with other TMS metrics) may benefit from targeted rehabilitation programs even in the chronic stage after SCI (47). These studies showed that the TMS can be used to study corticomotor reorganization with changes in cortical muscle representations following SCI.

#### Intracortical circuits

Paired-pulse TMS can be used to study the intracortical circuits after SCI. Paired-pulse TMS paradigms involve delivering a conditioning TMS pulse before a test TMS pulse (see review by Chen, 2004) (82) (see Table 6 for common protocols). Some of these measures such as short-interval intracortical inhibition (SICI), long-interval intracortical inhibition (LICI), intracortical facilitation (ICF), and short-interval intracortical facilitation (SICF) have been used in individuals with SCI to study changes at the cortical level (48, 83–85). A study found reduced SICI in the tibialis anterior muscle in individuals with incomplete chronic SCI (C3–T12) compared to healthy controls (83). Moreover, SICI recorded from the first dorsal interossei muscles was greater than that from the tibialis anterior muscle (83). Another study reported reduced SICI and LICI in the flexor carpi radialis muscle in individuals with incomplete chronic SCI (C3–C7 level) (48). However, when individuals with motor thresholds similar to healthy controls were included to control for the differences in motor threshold, SICI was not different between the groups (48) but LICI remained different, suggesting that changes in LICI were not due to excitability differences (48). Another study reported distinct modulation of different SICF peaks following chronic incomplete SCI with reduced magnitude for all SICF peaks, delayed latencies for second and third peaks, and a longer duration for only the third peak in individuals with chronic incomplete SCI (85). Using various TMS coil orientations to induce current in different directions, a study demonstrated that corticospinal responses elicited by targeting different cortical circuits are affected to varying extent by SCI (86). In a follow-up study that involved TMS during precision and power grips, the authors found these cortical circuits were engaged to a different extent in individuals with and without SCI (87). These studies show that TMS can be used to study the changes in the intracortical excitatory and inhibitory networks in individuals with SCI.

**Table 6:** Protocols for the paired-pulse TMS paradigms used in individuals with spinal cord injury.

TMS measure	Reference	Muscle	Conditioning pulse intensity	Test pulse intensity	Inter-stimulus interval (ms)	Changes after spinal cord injury
SICI	Mi et al., 2015 (48)	Flexor Carpi Radialis (active 15%–20% MVC)	60% AMT to 110% AMT	MEP_halfmax_ [defined as the MSO required to elicit a rectified area equal to half of the maximum]	3	Decreased inhibition when AMT not matched; no differences in inhibition when matched for AMT
SICI	Saturno et al., 2008 (84)	Extensor Digitorum Communis	80% RMT	120% RMT	2, 3, 5	Absence of inhibition; no control group
SICI	Roy et al., 2011 (83)	Tibialis Anterior (active 15%–20% MVC)	60% AMT to 110% AMT	½ * MEP_max_ [The TS intensity was set to the sensitive portion of the recruitment curve (i.e., producing test MEPs near ½ MEPmax).]	3	Inhibition in SCI group seen only at CS of 80% AMT v/s inhibition seen at 60%–90% AMT in healthy controls
LICI	Mi et al., 2015 (48)	Flexor Carpi Radialis (active 15%–20% MVC)	90% AMT to 130% AMT	MEP_halfmax_ [defined as the MSO required to elicit a rectified area equal to half of the maximum]	150	Decreased in individuals with SCI with and without AMT matching
ICF	Saturno et al., 2008 (84)	Extensor Digitorum Communis	80% RMT	120% RMT	10, 15	Facilitation seen; no control group

AMT, active motor threshold; ICF, intracortical facilitation; SICI, short-interval intracortical inhibition; LICI, long-interval intracortical inhibition; MEP, motor evoked potential; MSO, maximum stimulator output; ms, milliseconds; MVC, maximum voluntary contraction; RMT, resting motor threshold; TMS, transcranial magnetic stimulation; TS, test stimulus.

### Discriminative and predictive ability, and clinical correlations of TMS measures

TMS-based measurement can differentiate between individuals with different extent of motor impairments. For example, a study found that individuals with greater severity of cervical SCI had smaller abductor digiti minimi MEP amplitudes (14). In addition, the upper extremity motor scores were different between the five categories (Abolished, Reappearing, Inconsistent, Mildly deteriorated, or Severely deteriorated) based on the consistency of MEP (14). In individuals with consistent MEPs, the MEP amplitude correlated with the upper extremity motor scores (14). Using SICF, a study reported correlations between the upper extremity reaction time (latency and its variability) and amplitudes and latencies of later peaks of MEPs from the first dorsal interossei muscle (85). Another study found negative correlations between the cross-sectional area of the spinal cord at the cervical level and TMS-based motor threshold and cSP duration (40). These results suggest that greater atrophy of the cervical cord is associated with reduced corticospinal excitability and prolonged inhibition (40). In the lower extremities, the MEP amplitude of the tibialis anterior muscle showed good correlation with better performance in clinical measures of gait including the Walking Index for Spinal Cord Injury, the Timed-Up and Go, the 6-Min Walking Test, and the maximal treadmill gait speed (39). In addition, smaller MEP amplitudes were associated with greater atrophy in the lateral–ventral quadrant of the spinal cord on the more impaired side (39). TMS-based assessment of residual connectivity after SCI has also shown to be correlated with spasticity in the lower extremity muscles (88, 89). TMS metrics are also sensitive to neurophysiological changes following rehabilitation training. For example, rehabilitation training was associated with anterior shift in COG, along with an increase in the map area and volume for the biceps brachii muscle in an individual with chronic complete C6 SCI (80). Moreover, voluntary contraction-related decrease in motor map area of the first dorsal interosseous muscle was associated with the sensory deficits in the hand, and 10 min of vibration over hand muscle-tendon increased the motor map area during voluntary contraction (81). Improvements in upper extremity strength following intensive training has shown to correlate with excitability and motor map changes in muscles with different extent of impairments following SCI (90). Similarly, intensive locomotor training led to increased MEP amplitudes and the slope of the recruitment curve in individuals with SCI (91).

## Conclusion and future directions

Currently, clinical assessments are the main forms of evaluations during acute and chronic phases of SCI rehabilitation. ISNCSCI is the most standardized and commonly used clinical assessment following SCI. There are over 30 other outcome measures that have been validated and recommended for use in SCI. In addition, there are clinical prediction rules that combine clinical features, such as demographics, symptoms, physical examination findings, imaging results, and assessment scores for better prediction of outcomes. Clinical assessments are valuable in understanding recovery profiles and functional gains over the course of rehabilitation. However, they are subjective and do not provide information about the neurological processes underlying SCI, which limits their prognostication value.

TMS is an objective neurophysiological assessment tool and its different measures offer extensive information on corticomotor function. TMS offers a better insight into residual corticomotor connectivity, which may go undetected with the clinical scores of sensory and motor assessment. Due to this, TMS can be used to identify discomplete SCI. The TMS-based insights into neurophysiology can also be combined with the anatomical findings that are obtained using other techniques, such as diffusor tensor imaging or spinal tractography for more comprehensive understanding of corticomotor impairments and residual connectivity. However, whether the individuals with discomplete injuries would benefit from a different course of rehabilitation than those with complete injuries is a topic that needs to be investigated by future studies. TMS-based investigation of MEP and cSP characteristics (amplitude, latency, duration) provides insights into excitatory and inhibitory pathways following SCI. TMS-based motor maps allow study of corticomotor reorganization following SCI. Displacement of motor map COG or changes in its size following SCI or rehabilitation reflect neuroplastic changes following SCI. Paired-pulse TMS measures (SICI, LICI, ICF, SICF) help understand the compensatory reorganization of the cortical circuits following SCI. TMS can be combined with peripheral stimulation to study the central motor conduction time and modulation of spinal reflexes, that can be used for more advanced diagnostic and treatment purposes. Different TMS-based measures are able to differentiate between individuals with different severity levels of SCI, and correlate with the extent of injury and clinical scores. Lastly, TMS requires lesser time and training, has a higher temporal resolution and is more cost-effective than many neuroimaging techniques. Based on the above discussed advantages, TMS can be used to supplement clinical assessments in acute and chronic stages of SCI.

Future studies are needed to strengthen the use of TMS for clinical assessments in individuals with SCI. There are discrepancies in the findings of TMS studies, for example, delayed (41, 45) vs. non-delayed (42, 47) MEP latencies, smaller (14, 48) vs. larger (67) MEP amplitudes, absent (75) vs. similar (44) vs. prolonged (40) cSP, smaller (47) vs. large motor maps (81). The factors leading to these discrepancies need to be addressed. Methodological differences (e.g., sample size, target muscles, active vs. resting muscle state, extent of background contraction, type of TMS coils, coil orientation, stimulation intensities, definitions for MEP/cSP onset and offset) can contribute to these differences. Some of the differences can be addressed by standardizing the protocols. For example, selecting and reporting the appropriate coil orientation to target different cortical pathways (86, 87). Similarly, selecting suitable coil type for targeting of different muscles, for example double-cone (39, 92) or specialized batwing coil (54) for the lower extremity muscles and figure-of-eight coil (60) for the upper extremity muscles. Issues related to the study population may be more challenging to address. For example, early fatigue in muscles affected by SCI may make it challenging to maintain sustained background contraction throughout the testing, especially in severely impaired muscles. Antispastic medications (e.g., baclofen) that are commonly prescribed in individuals with SCI may affect TMS measures (93, 94). It has been suggested that the effects of antispastic medications on MEP are overridden by the volitional excitatory drive when testing the actively contracting muscle (49), but this needs to be tested with more extensive studies. Peripheral afferents input affect cortical excitability, and hence TMS measures (54, 59). Therefore, it is important to understand that changes in TMS measures may not always reflect changes in corticomotor transmission, but may also result from other sources such as afferent-based modulation of cortical pathways. Lower motor neuron lesions influence TMS measures and should be taken into consideration by use of F-waves (14), H-reflexes or lower motor neuron integrity tests (95). The psychometric properties of any technique are population specific, and currently only a few studies have investigated the psychometric properties of TMS-based measures in individuals with SCI (44, 96, 97). The smallest detectable change (SDC; the smallest change that is above the inherent measurement error and can be reliably detected) of TMS-based measures in proximal arm muscles is typically high for individuals with SCI (96). Changes in TMS measures should exceed these high SDC values to be considered as real change, which makes it challenging to use TMS measures as individual biomarkers. There is upcoming work addressing the feasibility and relevance of TMS-based assessment after SCI in the rehabilitation settings, and validating their use (along with imaging assessments) as predictive markers (98). The clinically meaningful difference of TMS-based metrics have yet to be established for individuals with SCI.

In conclusion, TMS allows detection of residual corticospinal connectivity following SCI. The measurement of MEP, cSP, cortical muscle representations, and intracortical circuits allows better understanding of the neurophysiology of corticomotor impairments and recovery following SCI. Due to its objectivity and ability to probe into neurophysiological mechanisms, TMS can supplement clinical assessments after SCI and help in devising targeted and individualized therapies for movement recovery. Studies with larger sample size and standardized protocols are needed to improve consistency in TMS-based findings in individuals with SCI. More research is needed to establish the psychometric properties of TMS-based measurements in the SCI population.

## References

[1] Jain NB Ayers GD Peterson EN Harris MB Morse L O’Connor KC Traumatic spinal cord injury in the United States, 1993-2012. JAMA. (2015) 313(22):2236–43. 10.1001/jama.2015.6250 26057284 PMC4712685

[2] Lasfargues JE Custis D Morrone F Cars well J Nguyen T. A model for estimating spinal cord injury prevalence in the United States. Spinal Cord. (1995) 33(2):62–8. 10.1038/sc.1995.16 7753569

[3] National Spinal Cord Injury Statistical Center. Spinal cord injury facts and figures at a glance. Birmingham, AL (2020). https://www.nscisc.uab.edu/Public/Facts%20and%20Figures%202020.pdf

[4] Anderson KD. Targeting recovery: priorities of the spinal cord-injured population. J Neurotrauma. (2004) 21(10):1371–83. 10.1089/neu.2004.21.1371 15672628

[5] Simpson LA Eng JJ Hsieh JTC Wolfe DL. The health and life priorities of individuals with spinal cord injury: a systematic review. J Neurotrauma. (2012) 29(8):1548–55. 10.1089/neu.2011.2226 22320160 PMC3501530

[6] Côté MP Murray M Lemay MA. Rehabilitation strategies after spinal cord injury: inquiry into the mechanisms of success and failure. J Neurotrauma. (2017) 34(10):1841–57. 10.1089/neu.2016.4577 27762657 PMC5444418

[7] Pizzolato C Gunduz MA Palipana D Wu J Grant G Hall S Non-invasive approaches to functional recovery after spinal cord injury: therapeutic targets and multimodal device interventions. Exp Neurol. (2021) 339:113612. 10.1016/j.expneurol.2021.113612 33453213

[8] Kirshblum S Snider B Eren F Guest J. Characterizing natural recovery after traumatic spinal cord injury. J Neurotrauma. (2021) 38(9):1267–84. 10.1089/neu.2020.7473 33339474 PMC8080912

[9] Scivoletto G Tamburella F Laurenza L Torre M Molinari M. Who is going to walk? A review of the factors influencing walking recovery after spinal cord injury. Front Hum Neurosci. (2014) 8:1–11. 10.3389/fnhum.2014.00141 24659962 PMC3952432

[10] Chandrasekaran S Bhagat N Ramdeo R Sharma PD Stein A Harkema SJ Targeted transcutaneous cervical spinal cord stimulation promotes upper limb recovery in spinal cord and peripheral nerve injury. medRxiv. (2022). 10.1101/2022.02.15.2226911535898335

[11] Rowald A Komi S Demesmaeker R Baaklini E Hernandez-Charpak SD Paoles E Activity-dependent spinal cord neuromodulation rapidly restores trunk and leg motor functions after complete paralysis. Nat Med. (2022) 28(2):260–71. 10.1038/s41591-021-01663-5 35132264

[12] Hubli M Kramer JLK Jutzeler CR Rosner J Furlan JC Tansey KE Application of electrophysiological measures in spinal cord injury clinical trials: a narrative review. Spinal Cord. (2019) 57(11):909–23. 10.1038/s41393-019-0331-z 31337870

[13] Cadotte DW Fehlings MG. Will imaging biomarkers transform spinal cord injury trials? Lancet Neurol. (2013) 12(9):843–4. 10.1016/S1474-4422(13)70157-1 23827393

[14] Petersen JA Spiess M Curt A Weidner N Rupp R Abel R Upper limb recovery in spinal cord injury: involvement of central and peripheral motor pathways. Neurorehabil Neural Repair. (2017) 31(5):432–41. 10.1177/1545968316688796 28132610

[15] Curt A Dietz V. Electrophysiological recordings in patients with spinal cord injury: significance for predicting outcome. Spinal Cord. (1999) 37(3):157–65. 10.1038/sj.sc.3100809 10213324

[16] Cook CE. Potential pitfalls of clinical prediction rules. J Man Manip Ther. (2008) 16(2):69–71. 10.1179/106698108790818477 19119389 PMC2565112

[17] Nater A Fehlings MG. Clinical prediction rules: the importance of the validation phase. Spine J. (2017) 17(10):1393–6. 10.1016/j.spinee.2017.06.002 28947011

[18] Engel-Haber E Zeilig G Haber S Worobey L Kirshblum S. The effect of age and injury severity on clinical prediction rules for ambulation among individuals with spinal cord injury. Spine J. (2020) 20(10):1666–75. 10.1016/j.spinee.2020.05.551 32502654

[19] Ariji Y Hayashi T Ideta R Koga R Murai S Towatari F A prediction model of functional outcome at 6 months using clinical findings of a person with traumatic spinal cord injury at 1 month after injury. Spinal Cord. (2020) 58(11):1158–65. 10.1038/s41393-020-0488-5 32444638

[20] Facchinello Y Beauséjour M Richard-Denis A Thompson C Mac-Thiong J-M. Use of regression tree analysis for predicting the functional outcome after traumatic spinal cord injury. J Neurotrauma. (2021) 38(9):1285–91. 10.1089/neu.2017.5321 29065782

[21] van Middendorp JJ Hosman AJ Donders AR Pouw MH Ditunno JF Jr. Curt A A clinical prediction rule for ambulation outcomes after traumatic spinal cord injury: a longitudinal cohort study. Lancet. (2011) 377(9770):1004–10. 10.1016/S0140-6736(10)62276-3 21377202

[22] Wilson JR Grossman RG Frankowski RF Kiss A Davis AM Kulkarni A V A clinical prediction model for long-term functional outcome after traumatic spinal cord injury based on acute clinical and imaging factors. J Neurotrauma. (2012) 29(13):2263–71. 10.1089/neu.2012.2417 22709268 PMC3430477

[23] Hicks KE Zhao Y Fallah N Rivers CS Noonan VK Plashkes T A simplified clinical prediction rule for prognosticating independent walking after spinal cord injury: a prospective study from a Canadian multicenter spinal cord injury registry. Spine J. (2017) 17(10):1383–92. 10.1016/j.spinee.2017.05.031 28716636

[24] Tetreault L Le D Côté P Fehlings M. The practical application of clinical prediction rules: a commentary using case examples in surgical patients with degenerative cervical myelopathy. Glob Spine J. (2015) 5(6):457–65. 10.1055/s-0035-1567838 PMC4671907 26682095

[25] Eng J Teasell R Miller W Wolfe D Townson A Aubut J-A Spinal cord injury rehabilitation evidence: method of the SCIRE systematic review. Top Spinal Cord Inj Rehabil. (2007) 13(1):1–10. 10.1310/sci1301-1 22767989 PMC3389040

[26] Kirshblum S Waring W. Updates for the international standards for neurological classification of spinal cord injury. Phys Med Rehabil Clin. (2014) 25(3):505–17. 10.1016/j.pmr.2014.04.001 25064785

[27] Kirshblum S Snider B Rupp R Read MS. Updates of the international standards for neurologic classification of spinal cord injury: 2015 and 2019. Phys Med Rehabil Clin. (2020) 31(3):319–30. 10.1016/j.pmr.2020.03.005 32624097

[28] Rupp R Biering-Sørensen F Burns SP Graves DE Guest J Jones L International standards for neurological classification of spinal cord injury. Top Spinal Cord Inj Rehabil. (2021) 27(2):1–22. 10.46292/sci2702-1 34108832 PMC8152171

[29] ASIA and ISCoS International Standards Committee. The 2019 revision of the international standards for neurological classification of spinal cord injury (ISNCSCI)-what’s new? Spinal Cord. (2019) 57(10):815–7. 10.1038/s41393-019-0350-9 31530900

[30] Kirshblum S Schmidt Read M Rupp R. Classification challenges of the 2019 revised international standards for neurological classification of spinal cord injury (ISNCSCI). Spinal Cord. (2022) 60(1):11–7. 10.1038/s41393-021-00648-y 34088981 PMC8737267

[31] Tanadini LG Hothorn T Jones LAT Lammertse DP Abel R Maier D Toward inclusive trial protocols in heterogeneous neurological disorders: prediction-based stratification of participants with incomplete cervical spinal cord injury. Neurorehabil Neural Repair. (2015) 29(9):867–77. 10.1177/1545968315570322 25644238

[32] Kalsi-Ryan S Wilson J Yang JM Fehlings MG. Neurological grading in traumatic spinal cord injury. World Neurosurg. (2014) 82(3–4):509–18. 10.1016/j.wneu.2013.01.007 23298673

[33] van Middendorp JJ Hosman AJ Pouw MH Van de Meent H. Is determination between complete and incomplete traumatic spinal cord injury clinically relevant? Validation of the ASIA sacral sparing criteria in a prospective cohort of 432 patients. Spinal Cord. (2009) 47(11):809–16. 10.1038/sc.2009.44 19468282

[34] Schuld C Franz S Van Hedel HJA Moosburger J Maier D Abel R International standards for neurological classification of spinal cord injury: classification skills of clinicians versus computational algorithms. Spinal Cord. (2015) 53(4):324–31. 10.1038/sc.2014.221 25487243

[35] Osunronbi T Sharma H. International standards for neurological classification of spinal cord injury: factors influencing the frequency, completion and accuracy of documentation of neurology for patients with traumatic spinal cord injuries. Eur J Orthop Surg Traumatol. (2019) 29(8):1639–48. 10.1007/s00590-019-02502-7 31324967 PMC6851215

[36] Armstrong AJ Clark JM Ho DT Payne CJ Nolan S Goodes LM Achieving assessor accuracy on the international standards for neurological classification of spinal cord injury. Spinal Cord. (2017) 55(11):994–1001. 10.1038/sc.2017.67 28631745

[37] Marino RJ Ditunno JF Jr. Donovan WH Maynard F Jr. Neurologic recovery after traumatic spinal cord injury: data from the model spinal cord injury systems. Arch Phys Med Rehabil. (1999) 80(11):1391–6. 10.1016/S0003-9993(99)90249-6 10569432

[38] Barker AT Jalinous R Freeston IL. Non-invasive magnetic stimulation of human motor cortex. Lancet. (1985) 325(8437):1106–7. 10.1016/S0140-6736(85)92413-4 2860322

[39] Barthélemy D Willerslev-Olsen M Lundell H Biering-Sørensen F Nielsen JB. Assessment of transmission in specific descending pathways in relation to gait and balance following spinal cord injury. Prog Brain Res. (2015) 218:79–101. 10.1016/bs.pbr.2014.12.012 25890133

[40] Freund P Rothwell J Craggs M Thompson AJ Bestmann S. Corticomotor representation to a human forearm muscle changes following cervical spinal cord injury. Eur J Neurosci. (2011) 34(11):1839–46. 10.1111/j.1460-9568.2011.07895.x 22082003

[41] Squair JW Bjerkefors A Inglis JT Lam T Carpenter MG. Cortical and vestibular stimulation reveal preserved descending motor pathways in individuals with motorcomplete spinal cord injury. J Rehabil Med. (2016) 48(7):589–96. 10.2340/16501977-2101 27292455

[42] Bjerkefors A Squair JW Chua R Lam T Chen Z Carpenter MG. Assessment of abdominal muscle function in individuals with motor-complete spinal cord injury above T6 in response to transcranial magnetic stimulation. J Rehabil Med. (2015) 47(2):138–46. 10.2340/16501977-1901 25502735

[43] Williams AMM Eginyan G Deegan E Chow M Carpenter MG Lam T. Residual innervation of the pelvic floor muscles in people with motor-complete spinal cord injury. J Neurotrauma. (2020) 37(21):2320–31. 10.1089/neu.2019.6908 32718211

[44] Sfreddo HJ Wecht JR Alsalman OA Wu Y-K Harel NY. Duration and reliability of the silent period in individuals with spinal cord injury. Spinal Cord. (2021) 59(8):885–93. 10.1038/s41393-021-00649-x 34099882

[45] Smith HC Savic G Frankel HL Ellaway PH Maskill DW Jamous MA Corticospinal function studied over time following incomplete spinal cord injury. Spinal Cord. (2000) 38(5):292–300. 10.1038/sj.sc.3100994 10822402

[46] Davey NJ Smith HC Wells E Maskill DW Savic G Ellaway PH Responses of thenar muscles to transcranial magnetic stimulation of the motor cortex in patients with incomplete spinal cord injury. J Neurol Neurosurg Psychiatry. (1998) 65(1):80–7. 10.1136/jnnp.65.1.80 9667566 PMC2170166

[47] Cortes M Thickbroom GW Elder J Rykman A Valls-Sole J Pascual-Leone A The corticomotor projection to liminally-contractable forearm muscles in chronic spinal cord injury: a transcranial magnetic stimulation study. Spinal Cord. (2017) 55(4):362–6. 10.1038/sc.2016.161 27995943

[48] Mi YP Bailey AZ Nelson AJ. Short- and long-intracortical inhibition in incomplete spinal cord injury. Can J Neurol Sci. (2015) 43(1):183–91. 10.1017/cjn.2015.310 26786645

[49] McKay WB Stokic DS Dimitrijevic MR. Assessment of corticospinal function in spinal cord injury using transcranial motor cortex stimulation: a review. J Neurotrauma. (1997) 14(8):539–48. 10.1089/neu.1997.14.539 9300564

[50] Hayes KC Allatt RD Wolfe DL Kasai T Hsieh J. Reinforcement of subliminal flexion reflexes by transcranial magnetic stimulation of motor cortex in subjects with spinal cord injury. Electroencephalogr Clin Neurophysiol. (1992) 85(2):102–9. 10.1016/0168-5597(92)90075-M 1373362

[51] Vasquez N Balasubramaniam V Kuppuswamy A Knight S Susser J Gall A The interaction of cortico-spinal pathways and sacral sphincter reflexes in subjects with incomplete spinal cord injury: a pilot study. Neurourol Urodyn. (2015) 34(4):349–55. 10.1002/nau.22554 25867009

[52] Sherwood AM Dimitrijevic MR Barry McKay W. Evidence of subclinical brain influence in clinically complete spinal cord injury: discomplete SCI. J Neurol Sci. (1992) 110(1–2):90–8. 10.1016/0022-510X(92)90014-C 1506875

[53] Alexeeva N Broton JG Calancie B. Latency of changes in spinal motoneuron excitability evoked by transcranial magnetic brain stimulation in spinal cord injured individuals. Electroencephalogr Clin Neurophysiol. (1998) 109(4):297–303. 10.1016/S0924-980X(98)00021-6 9751291

[54] Roy FD Yang JF Gorassini MA. Afferent regulation of leg motor cortex excitability after incomplete spinal cord injury. J Neurophysiol. (2010) 103(4):2222–33. 10.1152/jn.00903.2009 20181733

[55] Cariga P Catley M Nowicky A V Savic G Ellaway PH Davey NJ. Segmental recording of cortical motor evoked potentials from thoracic paravertebral myotomes in complete spinal cord injury. Spine. (2002) 27(13):1438–43. 10.1097/00007632-200207010-00013 12131743

[56] Oudega M Perez MA. Corticospinal reorganization after spinal cord injury. J Physiol. (2012) 590(16):3647–63. 10.1113/jphysiol.2012.233189 22586214 PMC3476625

[57] Chen R Cros D Curra A Di Lazzaro V Lefaucheur JP Magistris MR The clinical diagnostic utility of transcranial magnetic stimulation: report of an IFCN committee. Clin Neurophysiol. (2008) 119(3):504–32. 10.1016/j.clinph.2007.10.014 18063409

[58] Udupa K Chen R. Chapter 31 - Central motor conduction time. In: Lozano AM, Hallett M, editors. Handbook of clinical neurology. 1st edn. Vol 116. Elsevier B.V. (2013). p. 375–86. 10.1016/B978-0-444-53497-2.00031-0. [Epub ahead of print] 24112910

[59] Bailey AZ Mi YP Nelson AJ. Short-latency afferent inhibition in chronic spinal cord injury. Transl Neurosci. (2015) 6(1):235–43. 10.1515/tnsci-2015-0025 28123808 PMC4936633

[60] Jo HJ Perez MA. Corticospinal-motor neuronal plasticity promotes exercise-mediated recovery in humans with spinal cord injury. Brain. (2020) 143(5):1368–82. 10.1093/brain/awaa052 32355959 PMC7534104

[61] Bunday KL Urbin MA Perez MA. Potentiating paired corticospinal-motoneuronal plasticity after spinal cord injury. Brain Stimul. (2018) 11(5):1083–92. 10.1016/j.brs.2018.05.006 29848448

[62] Tolmacheva A Savolainen S Kirveskari E Brandstack N Mäkelä JP Shulga A. Paired associative stimulation improves hand function after non-traumatic spinal cord injury: a case series. Clin Neurophysiol Pract. (2019) 4:178–83. 10.1016/j.cnp.2019.07.002 31886442 PMC6921158

[63] Shulga A Lioumis P Kirveskari E Savolainen S Mäkelä JP. A novel paired associative stimulation protocol with a high-frequency peripheral component: a review on results in spinal cord injury rehabilitation. Eur J Neurosci. (2021) 53(9):3242–57. 10.1111/ejn.15191 33738876

[64] Fok KL Kaneko N Sasaki A Nakagawa K Nakazawa K Masani K. Motor point stimulation in spinal paired associative stimulation can facilitate spinal cord excitability. Front Hum Neurosci. (2020) 14:1–12. 10.3389/fnhum.2020.593806 33328940 PMC7729006

[65] Cash RFH Jegatheeswaran G Ni Z Chen R. Modulation of the direction and magnitude of hebbian plasticity in human motor cortex by stimulus intensity and concurrent inhibition. Brain Stimul. (2017) 10(1):83–90. 10.1016/j.brs.2016.08.007 27615792

[66] Hebb DO. The organization of behavior: A neuropsychological theory. New York: Psychology Press (2005).

[67] Nardone R. Enhanced motor cortex excitability after spinal cord injury. Neural Regen Res. (2015) 10(12):1943–4. 10.4103/1673-5374.172312 26889179 PMC4730815

[68] Davey NJ Smith HC Savic G Maskill DW Ellaway PH Frankel HL. Comparison of input-output patterns in the corticospinal system of normal subjects and incomplete spinal cord injured patients. Exp Brain Res. (1999) 127(4):382–90. 10.1007/s002210050806 10480273

[69] Lotze M Laubis-Herrmann U Topka H. Combination of TMS and FMRI reveals a specific pattern of reorganization in M1 in patients after complete spinal cord injury. Restor Neurol Neurosci. (2006) 24(2):97–107. 16720945

[70] Inghilleri M Berardelli A Cruccu G Manfredi M. Silent period evoked by transcranial stimulation of the human cortex and cervicomedullary junction. J Physiol. (1993) 466(1):521–34. 8410704 PMC1175490

[71] Chen R Lozano AM Ashby P. Mechanism of the silent period following transcranial magnetic stimulation evidence from epidural recordings. Exp Brain Res. (1999) 128(4):539–42. 10.1007/s002210050878 10541749

[72] Fuhr P Agostino R Hallett M. Spinal motor neuron excitability during the silent period after cortical stimulation. Electroencephalogr Clin Neurophysiol. (1991) 81(4):257–62. 10.1016/0168-5597(91)90011-L 1714819

[73] Ziemann U Netz J Szelényi A Hömberg V. Spinal and supraspinal mechanisms contribute to the silent period in the contracting soleus muscle after transcranial magnetic stimulation of human motor cortex. Neurosci Lett. (1993) 156(1–2):167–71. 10.1016/0304-3940(93)90464-V 8414181

[74] Nardone R Golaszewski S Bergmann J Venturi A Prünster I Bratti A Motor cortex excitability changes following a lesion in the posterior columns of the cervical spinal cord. Neurosci Lett. (2008) 434(1):119–23. 10.1016/j.neulet.2008.01.038 18280657

[75] Shimizu T Hino T Komori T Hirai S. Loss of the muscle silent period evoked by transcranial magnetic stimulation of the motor cortex in patients with cervical cord lesions. Neurosci Lett. (2000) 286(3):199–202. 10.1016/S0304-3940(00)01125-3 10832019

[76] Brown A Martinez M. From cortex to cord: motor circuit plasticity after spinal cord injury. Neural Regen Res. (2019) 14(12):2054–62. 10.4103/1673-5374.262572 31397332 PMC6788232

[77] Jr WJ L Amassian VE Traad M Cadwell J. Focal magnetic coil stimulation reveals motor cortical system reorganized in humans after traumatic quadriplegia. Brain Res. (1990) 510(1):130–4. 10.1016/0006-8993(90)90738-W 2322837

[78] Topka H Cohen LG Cole RA Hallett M. Reorganization of corticospinal pathways following spinal cord injury. Neurology. (1991) 41(8):1276. 10.1212/WNL.41.8.1276 1866018

[79] Leao MTD Wiesinger L Ziemann U Tatagiba M Naros G. Rapid motor cortical reorganization following subacute spinal cord dysfunction. Brain Stimul. (2020) 13(3):783–5. 10.1016/j.brs.2020.01.014 32289708

[80] Hoffman LR Field-Fote EC. Cortical reorganization following bimanual training and somatosensory stimulation in cervical spinal cord injury: a case report. Phys Ther. (2007) 87(2):208–23. 10.2522/ptj.20050365 17213410

[81] Tazoe T Perez MA. Abnormal changes in motor cortical maps in humans with spinal cord injury. J Physiol. (2021) 599(22):5031–45. 10.1113/JP281430 34192806 PMC9109877

[82] Chen R. Interactions between inhibitory and excitatory circuits in the human motor cortex. Exp Brain Res. (2004) 154(1):1–10. 10.1007/s00221-003-1684-1 14579004

[83] Roy FD Zewdie ET Gorassini MA. Short-interval intracortical inhibition with incomplete spinal cord injury. Clin Neurophysiol. (2011) 122(7):1387–95. 10.1016/j.clinph.2010.11.020 21295518

[84] Saturno E Bonato C Miniussi C Lazzaro V Callea L. Motor cortex changes in spinal cord injury: a TMS study. Neurol Res. (2008) 30(10):1084–5. 10.1179/174313208X332968 18768107

[85] Cirillo J Calabro FJ Perez MA. Impaired organization of paired-pulse TMS-induced I-waves after human spinal cord injury. Cereb Cortex. (2016) 26(5):2167–77. 10.1093/cercor/bhv048 25814508 PMC4830292

[86] Jo HJ Di Lazzaro V Perez MA. Effect of coil orientation on motor-evoked potentials in humans with tetraplegia. J Physiol. (2018) 596(20):4909–21. 10.1113/JP275798 29923194 PMC6187022

[87] Jo HJ Perez MA. Changes in motor-evoked potential latency during grasping after tetraplegia. J Neurophysiol. (2019) 122(4):1675–84. 10.1152/jn.00671.2018 30673355 PMC6843103

[88] Sangari S Kirshblum S Guest JD Oudega M Perez MA. Distinct patterns of spasticity and corticospinal connectivity following complete spinal cord injury. J Physiol. (2021) 599(19):4441–54. 10.1113/JP281862 34107068 PMC9053045

[89] Sangari S Perez MA. Imbalanced corticospinal and reticulospinal contributions to spasticity in humans with spinal cord injury. J Neurosci. (2019) 39(40):7872–81. 10.1523/JNEUROSCI.1106-19.2019 31413076 PMC6774405

[90] Potter-Baker KA Janini DP Lin Y-L Sankarasubramanian V Cunningham DA Varnerin NM Transcranial direct current stimulation (tDCS) paired with massed practice training to promote adaptive plasticity and motor recovery in chronic incomplete tetraplegia: a pilot study. J Spinal Cord Med. (2018) 41(5):503–17. 10.1080/10790268.2017.1361562 28784042 PMC6117576

[91] Thomas SL Gorassini MA. Increases in corticospinal tract function by treadmill training after incomplete spinal cord injury. J Neurophysiol. (2005) 94(4):2844–55. 10.1152/jn.00532.2005 16000519

[92] Kesar TM Stinear JW Wolf SL. The use of transcranial magnetic stimulation to evaluate cortical excitability of lower limb musculature: challenges and opportunities. Restor Neurol Neurosci. (2018) 36(3):333–48. 10.3233/RNN-170801 29758954 PMC6106786

[93] Inghilleri M Berardelli A Marchetti P Manfredi M. Effects of diazepam, baclofen and thiopental on the silent period evoked by transcranial magnetic stimulation in humans. Exp Brain Res. (1996) 109(3):467–72. 10.1007/BF00229631 8817277

[94] Barry MD Bunday KL Chen R Perez MA. Selective effects of baclofen on use-dependent modulation of GABAB inhibition after tetraplegia. J Neurosci. (2013) 33(31):12898–907. 10.1523/JNEUROSCI.1552-13.2013 23904624 PMC3728695

[95] Bryden AM Hoyen HA Keith MW Mejia M Kilgore KL Nemunaitis GA. Upper extremity assessment in tetraplegia: the importance of differentiating between upper and lower motor neuron paralysis. Arch Phys Med Rehabil. (2016) 97(6):S97–S104. 10.1016/j.apmr.2015.11.021 27233597

[96] Arora T Potter-Baker K O’Laughlin K Li M Wang X Cunningham D Measurement error and reliability of TMS metrics collected from biceps and triceps in individuals with chronic incomplete tetraplegia. Exp Brain Res. (2021) 239(10):1–13. 10.1007/s00221-021-06160-2 34374814

[97] Potter-Baker KA Janini DP Frost FS Chabra P Varnerin N Cunningham DA Reliability of TMS metrics in patients with chronic incomplete spinal cord injury. Spinal Cord. (2016) 54(11):980. 10.1038/sc.2016.47 27045553

[98] Huang YN Meftah EM Pion CH Mac-Thiong JM Cohen-Adad J Barthélemy D. Quantitative electrophysiological assessments as predictive markers of lower limb motor recovery after spinal cord injury: a pilot study with an adaptive trial design. Spinal Cord Ser Cases. (2022) 8(1):1–8. 10.1038/s41394-021-00473-8 35210402 PMC8873458

[99] Ni Z Chen R. Transcranial magnetic stimulation to understand pathophysiology and as potential treatment for neurodegenerative diseases. Transl Neurodegener. (2015) 4(1):1–12. 10.1186/s40035-015-0045-x 26579223 PMC4647804

[100] Catz A Itzkovich M Agranov E Ring H Tamir A. SCIM–Spinal cord independence measure: a new disability scale for patients with spinal cord lesions. Spinal Cord. (1997) 35(12):850–6. 10.1038/sj.sc.3100504 9429264

[101] Marino RJ Patrick M Albright W Leiby BE Mulcahey MJ Schmidt-Read M Development of an objective test of upper-limb function in tetraplegia: the capabilities of upper extremity test. Am J Phys Med Rehabil. (2012) 91(6):478–86. 10.1097/PHM.0b013e31824fa6cc 22469875

[102] Kalsi-Ryan S Curt A Verrier MC Fehlings MG. Development of the graded redefined assessment of strength, sensibility and prehension (GRASSP): reviewing measurement specific to the upper limb in tetraplegia. J Neurosurg Spine. (2012) 17(Suppl1):65–76. 10.3171/2012.6.AOSPINE1258 22985372

[103] Marino RJ Shea JA Stineman MG. The capabilities of upper extremity instrument: reliability and validity of a measure of functional limitation in tetraplegia. Arch Phys Med Rehabil. (1998) 79(12):1512–21. 10.1016/S0003-9993(98)90412-9 9862292

[104] Wuolle KS Van Doren CL Thrope GB Keith MW Peckham PH. Development of a quantitative hand grasp and release test for patients with tetraplegia using a hand neuroprosthesis. J Hand Surg Am. (1994) 19(2):209–18. 10.1016/0363-5023(94)90008-6 8201183

[105] Mulcahey MJ Smith BT Betz RR. Psychometric rigor of the grasp and release test for measuring functional limitation of persons with tetraplegia: a preliminary analysis. J Spinal Cord Med. (2004) 27(1):41–6. 10.1080/10790268.2004.11753729 15156936

[106] Tederko P Krasuski M Czech J Dargiel A Garwacka-Jodzis I Wojciechowska A. Reliability of clinical spasticity measurements in patients with cervical spinal cord injury. Ortop Traumatol Rehabil. (2007) 9(5):467–83. 18026067

[107] Benz EN Hornby TG Bode RK Scheidt RA Schmit BD. A physiologically based clinical measure for spastic reflexes in spinal cord injury. Arch Phys Med Rehabil. (2005) 86(1):52–9. 10.1016/j.apmr.2004.01.033 15640989

[108] Sollerman C Ejeskär A. Sollerman hand function test: a standardised method and its use in tetraplegic patients. Scand J Plast Reconstr Surg Hand Surg. (1995) 29(2):167–76. 10.3109/02844319509034334 7569815

[109] Fattal C Thery J-M Micallef J-P. Validation d’une grille de capacités motrices du tétraplégique opéré du ou des membres supérieurs. Ann Readapt Med Phys. (2004) 47(8):537–45. 10.1016/j.annrmp.2004.04.003 15465158

[110] Scivoletto G Tamburella F Laurenza L Torre M Molinari M Ditunno JF. Walking Index for spinal cord injury version II in acute spinal cord injury: reliability and reproducibility. Spinal Cord. (2014) 52(1):65–9. 10.1038/sc.2013.127 24145685

[111] van Hedel HJ Wirz M Dietz V. Assessing walking ability in subjects with spinal cord injury: validity and reliability of 3 walking tests. Arch Phys Med Rehabil. (2005) 86(2):190–6. 10.1016/j.apmr.2004.02.010 15706542

[112] Field-Fote EC Fluet GG Schafer SD Schneider EM Smith R Downey PA The spinal cord injury functional ambulation inventory (SCI-FAI). J Rehabil Med. (2001) 33(4):177–81. 10.1080/165019701750300645 11506216

[113] Lemay JF Nadeau S. Standing balance assessment in ASIA D paraplegic and tetraplegic participants: concurrent validity of the berg balance scale. Spinal Cord. (2010) 48(3):245–50. 10.1038/sc.2009.119 19773797

[114] Akpinar P Atici A Ozkan FU Aktas I Kulcu DG Kurt KN. Reliability of the spinal cord assessment tool for spastic reflexes. Arch Phys Med Rehabil. (2017) 98(6):1113–8. 10.1016/j.apmr.2016.09.119 27744026

[115] Itzkovich M Gelernter I Biering-Sorensen F Weeks C Laramee MT Craven BC The spinal cord independence measure (SCIM) version III: reliability and validity in a multi-center international study. Disabil Rehabil. (2007) 29(24):1926–33. 10.1080/09638280601046302 17852230

